# Dynapenic Abdominal Obesity and Adverse Health Effects in Middle-Aged and Older Adults: A Systematic Review and Meta-Analysis

**DOI:** 10.3390/healthcare13080916

**Published:** 2025-04-16

**Authors:** Li-Kuo Liu, Yu-Chen Su, Hsiao-Chi Tsai, Shu-Fang Chang

**Affiliations:** 1 Department of Family Medicine, Shin Kong Wu Ho-Su Memorial Hospital, Shilin District, Taipei City 111, Taiwan; nicole.liu@gmail.com; 2 Nursing Department, Hsin Sheng Junior College of Medical Care and Management, Longtan District, Taoyuan City 325, Taiwan; sue6227@hsc.edu.tw; 3 Cardinal Tien Hospital, Xindian District, New Taipei City 231, Taiwan; 4 School of Nursing, College of Nursing, National Taipei University of Nursing and Health Sciences, Beitou District, Taipei City 112, Taiwan

**Keywords:** dynapenic abdominal obesity, fall, disability, mortality, meta-analysis, systematic review

## Abstract

Background/Objectives: Dynapenic abdominal obesity is characterized by reduced muscle strength and abdominal fat accumulation and is associated with adverse health outcomes in older adults. Evidence from multiple studies conducted in different countries regarding these outcomes is inconsistent. This systematic review and meta-analysis evaluated the association between dynapenic abdominal obesity and adverse health outcomes—specifically falls, disability, and mortality—among middle-aged and older adults. Methods: A comprehensive search of Embase, PubMed, MEDLINE, CINAHL, and the Cochrane Library (from inception to December 2024) identified 12 eligible prospective cohort studies. Keywords included “older adults”, “elderly”, “older people”, “dynapenic abdominal obesity”, “fall”, “disability”, and “mortality”. A systematic review and meta-analysis was conducted using random-effects models, with subgroup analyses based on measurement methods and geographic regions. Results: Dynapenic abdominal obesity was significantly associated with increased risks of falls (hazard ratio = 1.82, 95% confidence interval: 1.04–3.17) and mortality (hazard ratio = 1.50, 95% confidence interval: 1.14–1.96). The subgroup analysis results indicated variations in outcomes across measurement criteria and countries. Dynapenic abdominal obesity was not significantly associated with disability risk (odds ratio = 0.92, 95% confidence interval: 0.18–4.54). Conclusions: Dynapenic abdominal obesity is associated with significantly increased fall and mortality risks in middle-aged and older adults. These findings underscore the need for targeted assessments and intervention strategies for high-risk groups.

## 1. Introduction

This introduction builds upon the objective outlined in the abstract, emphasizing the significance of examining dynapenic abdominal obesity in relation to adverse health outcomes in older adults. As the global population of older individuals rapidly increases (with more than 1 billion individuals currently aged 60 years or older), aging is becoming a pressing medical concern [1]. The World Health Organization [1] estimates that by 2100, the global population aged 60 years or older will increase from 900 million to 3.2 billion individuals. As individuals age, muscle strength declines even when normal muscle mass is maintained, a phenomenon termed dynapenia. The prevalence of dynapenia varies across countries. For example, one study conducted in the United Kingdom reported that among individuals aged 60 years or older, the prevalence of dynapenia was approximately 25% [2]. Another study conducted in Italy and involving 846 older adults aged 60 years or older suggested that the prevalence of dynapenia in this age group was approximately 33% [3]. Furthermore, the increasing prevalence of excess body weight and obesity, which affects nearly one-third of the global population, is associated with physical activity limitations, disabilities, and poor quality of life. Abdominal obesity, characterized by an increase in visceral adipose tissue, poses a greater risk of metabolic diseases than does peripheral obesity because of the distribution of fat around the central organs [3]. Waist circumference is often used as a measure of abdominal obesity. Muga et al. [4] analyzed the nutritional and health status of Taiwanese adults over three periods, revealing an increase in the prevalence of abdominal obesity from 27% to 47% between 1993 and 2016, with projected rates of 55.6% for men and 80.0% for women by 2023. These studies indicate a trend of increasing obesity, with nearly half of the individuals studied having abdominal obesity.

Dynapenic abdominal obesity refers to a condition in older adults in which muscle strength declines and abdominal fat accumulates [3]. In a cross-sectional study of 382 community-dwelling older adults in Brazil, Araújo et al. [5] uncovered a prevalence of dynapenic abdominal obesity of 10.73%. Alexandre et al. [6] analyzed 6173 older adults in the United Kingdom and Brazil, revealing a prevalence of dynapenic abdominal obesity of 7.2%. That study also conducted a 10-year follow-up to assess all-cause mortality and to compare the risk of mortality among individuals with dynapenia, abdominal obesity, and dynapenic abdominal obesity, observing the highest risk in those with dynapenic abdominal obesity (hazard ratio = 3.37, 95% confidence interval: 1.12–1.68). These findings suggest that the prevalence of dynapenic abdominal obesity ranges from 7.2% to 10.73% among older adults. Moreover, studies have demonstrated that dynapenic abdominal obesity is associated with a range of adverse health outcomes, including fractures, hospitalizations, disability, and mortality [7].

Studies have also emphasized the clinical importance of identifying dynapenic abdominal obesity early in older adults. Lin et al. [8] reported dietary and metabolic shifts in Taiwanese older adults that may contribute to increasing rates of abdominal obesity. Smith et al. [9] reported in a 2-year follow-up study that older adults with dynapenic abdominal obesity had a 47% higher risk of falls than did those without dynapenic abdominal obesity, underscoring the condition’s implications for mobility and safety. Veronese et al. [10] demonstrated a significant association between dynapenic abdominal obesity and increased risk of multimorbidity in aging populations. These findings suggest that dynapenic abdominal obesity should be considered a distinct geriatric phenotype of clinical importance.

Multiple studies have associated dynapenic abdominal obesity with increased risks of falls [2,11,12,13,14,15,16], disability [14], and mortality [17,18]; however, few studies have explored whether these risks vary across measurement methods, recruitment settings, or countries. Further investigation is warranted to address this gap in the literature.

### Aims

This study conducted a systematic review and meta-analysis to examine the association between dynapenic abdominal obesity and adverse health outcomes—specifically falls, disability, and mortality—among middle-aged and older adults (age ≥ 50 years) on the basis of evidence from multiple prospective cohort studies.

## 2. Methods

A meta-analysis was conducted to quantitatively synthesize data from eligible prospective cohort studies. Due to variability in reported outcomes across studies and the absence of pooled estimates in the existing literature, a meta-analytic approach was deemed essential to provide a clearer understanding of the risks associated with dynapenic abdominal obesity. To ensure methodological consistency, only original studies were included, and previously published systematic reviews or meta-analyses were excluded. We systematically searched the literature to investigate the associations between dynapenic abdominal obesity and adverse outcomes—specifically, falls, disability, and mortality. This study adhered to the Preferred Reporting Items for Systematic Reviews and Meta-Analyses (PRISMA) guidelines [19].

### 2.1. Data Sources and Search Strategy

We conducted a systematic review and meta-analysis to comprehensively synthesize and quantitatively evaluate evidence from prospective cohort studies investigating the associations of dynapenic abdominal obesity with adverse health outcomes. Additionally, we conducted subgroup analyses to identify potential variations among the included studies. Review articles, meta-analyses, and systematic reviews were excluded from our analysis to ensure the originality and accuracy of our findings. The researchers systematically searched the literature in Embase, PubMed, MEDLINE, CINAHL, and the Cochrane Library from database inception until December 2024. The search keywords were (“middle age” OR “older adults” OR “older people”) AND (“dynapenic abdominal obesity”) AND (“fall” OR “disability” OR “mortality”)

### 2.2. Inclusion and Exclusion Criteria

Articles were included if they (1) employed a prospective cohort design; (2) evaluated older adults with dynapenic abdominal obesity; (3) presented results as adjusted or unadjusted odds ratios or hazard ratios; (4) assessed differences in dynapenic abdominal obesity and negative outcomes; (5) included 95% CIs; and (6) were published in English and contained the full text. We excluded literature reviews, letters to editors, book chapters, experimental studies, and postgraduate theses (including master’s and PhD dissertations) to ensure that all included studies were peer-reviewed original research. This approach maintained methodological consistency and minimized potential sources of bias [20].

### 2.3. Data Extraction

Two independent reviewers conducted each stage of the selection and data extraction process, including title/abstract screening, full-text review, and data extraction using a standardized form. Extracted information included study authors, year, design, demographics, country, measurement criteria, primary outcomes, and statistical data. Discrepancies were resolved through discussion or by a third reviewer if needed.

### 2.4. Quality Assessment

The quality of the included prospective cohort studies was assessed using the Newcastle–Ottawa Scale (NOS), which assigns a maximum score of 9 based on three domains: selection, comparability, and outcome assessment. Studies with scores ≥7 were considered to have a low risk of bias, those with scores between 4 and 6 indicated a moderate risk, and scores <4 represented a high risk of bias (Table 1). To address heterogeneity, we conducted subgroup analyses based on geographic regions and measurement methods. These groupings were pre-defined according to variations in the literature and study characteristics, allowing clearer interpretation of differences across populations and methodologies.

### 2.5. Statistical Analysis

Odds ratios or hazard ratios were extracted from the included studies. If a study provided several odds ratios or hazard ratios (using different covariates), the odds ratios and hazard ratios that were the most adjusted were extracted. A random-effects model was employed to combine the odds ratios or hazard ratios and to account for variations in the true effect size across studies. The heterogeneity of effect sizes was assessed using the I^2^ statistic. All meta-analyses were conducted using Review Manager (RevMan) version 5.4 (The Cochrane Collaboration).

## 3. Results

### 3.1. Study Sample and Quality

**Figure 1:**
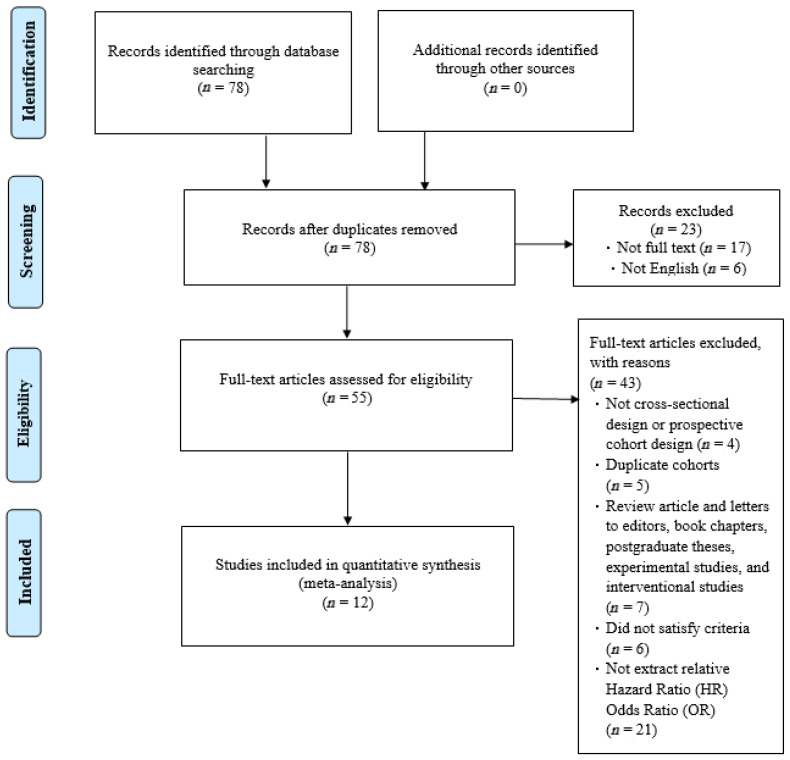
Preferred Reporting Items for Systematic Reviews and Meta-Analyses (PRISMA) flowchart.

**Figure 2:**
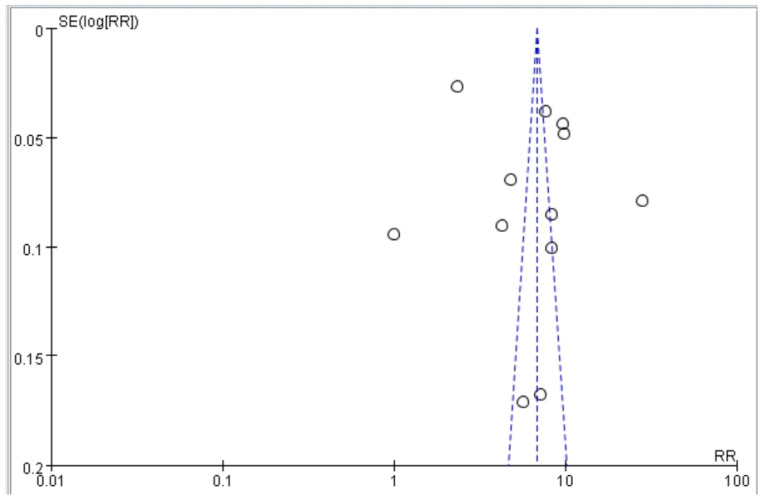
Funnel plot of studies assessing the association between dynapenic abdominal obesity and adverse outcomes (falls, disability, and mortality).

Our article selection process is illustrated in Figure 1. Initially, 78 studies were identified. Most were subsequently excluded on the basis of the following criteria: incomplete text, duplicate cohort, review article, failure to meet inclusion criteria, or relevant odds ratios or hazard ratios not available. After excluding these studies, 12 studies were selected for inclusion by both reviewers (Figure 1). The quality of the included studies was assessed using the NOS. Each study received a score of at least 8 (maximum score, 9), indicating high quality. Specifically, most studies exhibited clear selection criteria and reliable outcome. These results reduce the risk of bias in the synthesized findings. Funnel plots based on odds and hazard ratios were used to assess publication bias. The plots for falls, disability, and mortality were relatively symmetrical, suggesting low publication bias and supporting the robustness of the pooled estimates (Figure 2).

Table 2, Table 3 and Table 4 provide a summary of the key characteristics of the included studies.

The included studies collectively evaluated 34,066 individuals (Figure 1). Three studies were conducted in Brazil, two were conducted in England, two were conducted in China, one was conducted in Ireland, one was conducted in the United States, and three were conducted in Italy. Meanwhile, the final 12 studies include four that recruited only female participants and eight that included both sexes. Ten studies involved community-dwelling populations, while two included institutionalized older adults. Heterogeneity was evaluated using the I^2^ statistic and Cochran’s Q test, showing moderate to high heterogeneity across outcomes. This variation likely reflects differences in study populations, measurement methods, and settings. Subgroup analyses by geographic region and measurement criteria were conducted to further explore and clarify these differences.

### 3.2. Association Between Dynapenic Abdominal Obesity and Overall Fall Risk, Stratified by OR

**Figure 3:**
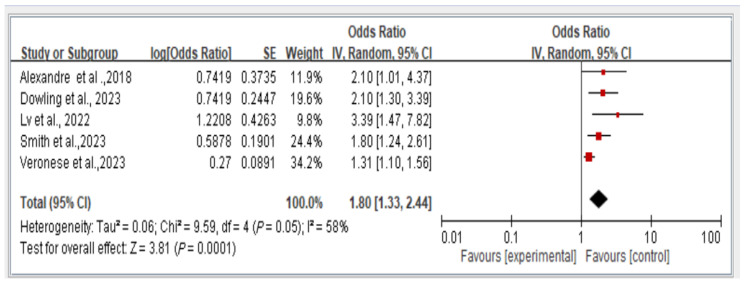
Association between dynapenic abdominal obesity and overall fall risk stratified by odds ratio [2,12,14,15,20]. This figure presents the results of the meta-analysis conducted in the present systematic review. The red squares represent the odds ratio estimates of individual studies, with size indicating study weight. The black diamond shows the overall pooled effect, where its center is the combined odds ratio and the width represents the 95% confidence interval.

The results reveal the overall fall risk across all five studies assessing this outcome. The findings indicate that individuals with dynapenic abdominal obesity had a higher risk of falls than did those without dynapenic abdominal obesity (odds ratio = 1.80, 95% CI = 1.33–2.44, *p* < 0.05; Figure 3).

### 3.3. Association Between Dynapenic Abdominal Obesity and Overall Fall Risk, Stratified by Hazard Ratio

**Figure 4:**
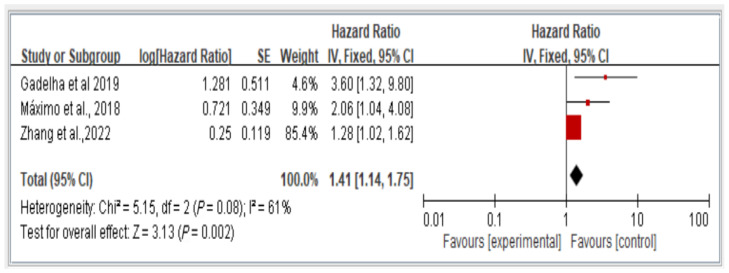
Association between dynapenic abdominal obesity and overall fall risk stratified by hazard ratio [11,13,16]. This figure presents the results of the meta-analysis conducted in the present systematic review. The red squares represent the odds ratio estimates of individual studies, with size indicating study weight. The black diamond shows the overall pooled effect, where its center is the combined odds ratio and the width represents the 95% confidence interval.

The results reveal the overall fall risk across all three studies assessing this variable. The findings indicate that individuals with dynapenic abdominal obesity had a higher risk of falls than did those without dynapenic abdominal obesity (hazard ratio = 1.41, 95% CI = 1.14–1.75, *p* < 0.05; Figure 4).

### 3.4. Subgroup Analysis of Measurement Criteria for the Risk of Falls, Stratified by Hazard Ratio

**Figure 5:**
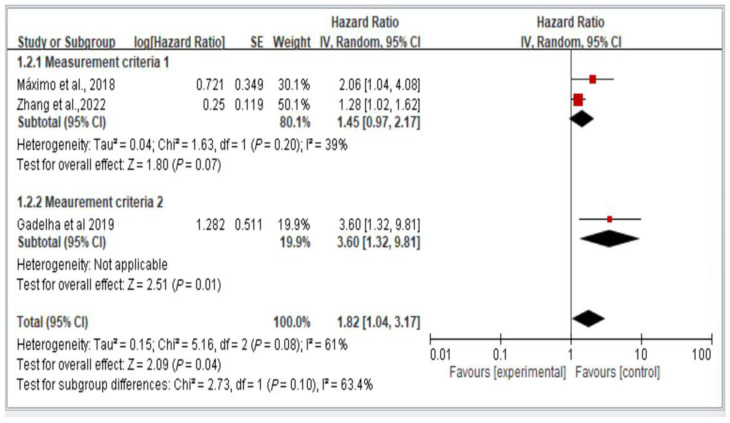
Subgroup analysis of different measurement criteria for falls, stratified by hazard ratio [11,13,16]. This figure presents the results from the meta-analysis conducted in the present systematic review. The red squares represent the odds ratio estimates of individual studies, with size indicating study weight. The black diamond shows the overall pooled effect, where its center is the combined odds ratio and the width represents the 95% confidence interval.

Figure 5 illustrates the two groups of measurement methods employed in the examined studies. Group 1 involved handgrip strength thresholds of <26 kg for men and <16 kg for women, as well as waist circumference thresholds of >102 cm for men and >88 cm for women. Group 2 encompassed all other measurement approaches. The results indicate the lack of a significant difference in fall risk among older individuals with dynapenic abdominal obesity across subgroups (hazard ratio = 1.82, 95% CI = 1.04–3.17, *p* < 0.05; Figure 5).

### 3.5. Subgroup Analysis of Fall Risk by Country (China vs. Brazil) Stratified by Hazard Ratio

**Figure 6:**
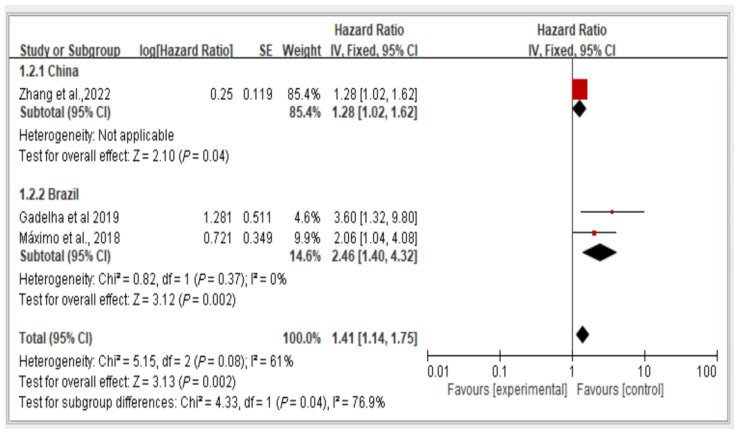
Fall risk in China and Brazil by hazard ratio [11,13,16]. This figure presents the results of the meta-analysis conducted in the present systematic review. The red squares represent the odds ratio estimates of individual studies, with size indicating study weight. The black diamond shows the overall pooled effect, where its center is the combined odds ratio and the width represents the 95% confidence interval.

We conducted a subgroup analysis of fall risk in the included studies on the basis of the country of publication. Figure 4 illustrates the fall risk among older adults with dynapenic abdominal obesity in China and Brazil stratified by hazard ratio. Among the included studies, one was conducted in China, and two were conducted in Brazil. The risk ratio between these two study groups was calculated and analyzed using a random-effects model. The results indicate a significant difference in fall risk among individuals with dynapenic abdominal obesity recruited from different countries. Specifically, older individuals with dynapenic abdominal obesity in China had a higher risk of falls than did those in Brazil (hazard ratio = 1.41, 95% CI = 1.14–1.75, *p* < 0.05; Figure 6).

### 3.6. Association Between Dynapenic Abdominal Obesity and Overall Disability Risk, Stratified by Odds Ratio

**Figure 7:**
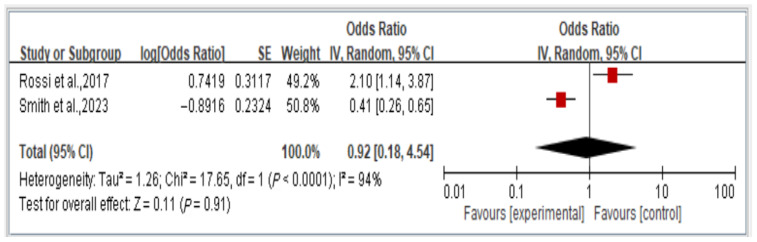
Relationship between dynapenic abdominal obesity and overall disability risk, stratified by odds ratio [14,18]. This figure presents the results of the meta-analysis conducted in the present systematic review. The red squares represent the odds ratio estimates of individual studies, with size indicating study weight. The black diamond shows the overall pooled effect, where its center is the combined odds ratio and the width represents the 95% confidence interval.

We analyzed the overall risk of disability associated with dynapenic abdominal obesity, stratified by OR across two studies. The findings indicate that the risk of disability is not significantly associated with dynapenic abdominal obesity (odds ratio = 0.92, 95% CI = 0.18–4.54, *p* > 0.05; Figure 7).

### 3.7. Association Between Dynapenic Abdominal Obesity and Overall Mortality Risk by Hazard Ratio

**Figure 8:**
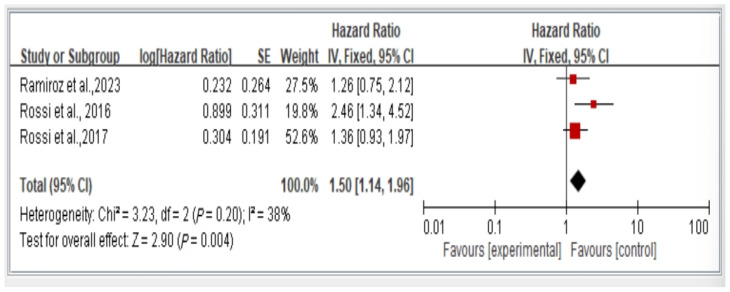
Relationship between dynapenic abdominal obesity and overall mortality risk stratified by hazard ratio [3,17,18]. This figure presents the results of the meta-analysis conducted in the present systematic review. The red squares represent the odds ratio estimates of individual studies, with size indicating study weight. The black diamond shows the overall pooled effect, where its center is the combined odds ratio and the width represents the 95% confidence interval.

The results from all studies on mortality risk show that individuals with dynapenic abdominal obesity have a higher mortality risk compared to those without dynapenic abdominal obesity (hazard ratio = 1.50, 95% CI = 1.14–1.96, *p* < 0.05) (Figure 8).

### 3.8. Subgroup Analysis of Measurement Criteria of Mortality Risk, Stratified by Hazard Ratio

**Figure 9:**
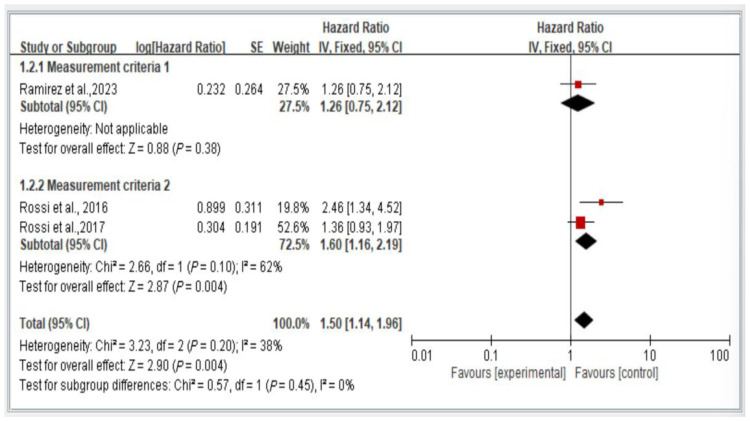
Subgroup analysis of mortality risk stratified by measurement criteria and hazard ratios [3,18,21]. This figure presents the results of the meta-analysis conducted in the present systematic review. The red squares represent the odds ratio estimates of individual studies, with size indicating study weight. The black diamond shows the overall pooled effect, where its center is the combined odds ratio and the width represents the 95% confidence interval.

Figure 9 illustrates the comparison of mortality risk among individuals with dynapenic abdominal obesity on the basis of different measurement criteria. We categorized the measurement criteria into two groups. Group 1 comprised studies evaluating handgrip strength (<26 kg for men and <16 kg for women) and waist circumference (>102 cm for men and >88 cm for women). Group 2 encompassed studies using all other measurement methods.

The results indicate a significant difference in mortality risk among individuals with dynapenic abdominal obesity assessed using different measurement criteria. Specifically, individuals classified using the measurement criteria of Group 1 exhibited a higher subsequent mortality risk than did those classified using the measurement criteria of Group 2 (hazard ratio = 1.50, 95% CI = 1.14–1.96, *p* < 0.05; Figure 9).

### 3.9. Subgroup Analysis of Mortality Risk in Italy and England, Stratified by Hazard Ratio

**Figure 10:**
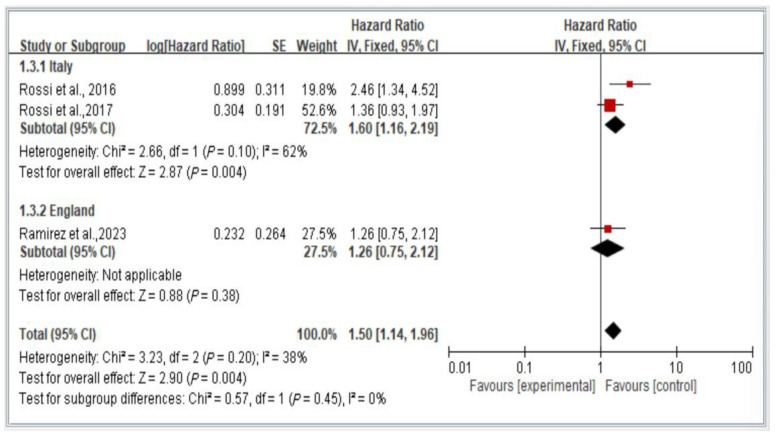
Subgroup analysis of mortality risk in Italy and England, stratified by hazard ratio [3,17,18]. This figure presents the results of the meta-analysis conducted in the present systematic review. The red squares represent the odds ratio estimates of individual studies, with size indicating study weight. The black diamond shows the overall pooled effect, where its center is the combined odds ratio and the width represents the 95% confidence interval.

Figure 10 depicts a comparison of mortality risk among individuals with dynapenic abdominal obesity across countries. The figure presents a comparison using a random-effects model of the results of two studies conducted in Italy and one conducted in England.

The results indicate a significant difference in subsequent mortality risk among individuals with dynapenic abdominal obesity across countries. Specifically, individuals in Italy exhibited a higher subsequent mortality risk than did individuals in England (hazard ratio = 1.50, 95% CI = 1.14–1.96, *p* < 0.05; Figure 10).

## 4. Discussion

The following discussion interprets the main findings presented in the results section, placing them in the context of existing literature and highlighting their clinical and research implications.

The present study evaluated 12 articles assessing a total of 34,066 individuals to examine the effects of dynapenic abdominal obesity on adverse health outcomes, such as falls and mortality, among middle-aged and older adults. The overall average follow-up time for tracking subsequent adverse health events was 6.70 years. Rossi et al. [21] suggested that middle-aged and older adults experiencing adverse health events due to dynapenia may exhibit slow progression, necessitating lengthy tracking periods for evaluation. Moreover, considerable differences exist in the range of follow-up times across the studies included in the present meta-analysis, with the longest follow-up time for fall risk being 14 years [21] and the shortest average follow-up time for fall risk being 1.5 years [9]. Smith et al. [9] emphasized that dynapenia in older individuals can lead to adverse health outcomes, imposing substantial burdens on individuals, their family caregivers, and society. Estimates of the annual cost in the United States alone of falls, disability, and hospitalizations among older adults range from US$11.8 billion to US$26.2 billion. Hence, health-care professionals must assess dynapenic abdominal obesity in older individuals early and provide care strategies to prevent or mitigate the occurrence of further adverse health events. Therefore, the differences in follow-up times across the studies included in the present meta-analysis may have varying effects on the health outcomes of older individuals with dynapenic abdominal obesity.

This study has several noteworthy features. Firstly, it represents the first systematic review and meta-analysis to investigate the relationship between dynapenic abdominal obesity and the occurrence of falls, disability, and mortality, thereby providing significant reference value to the current literature. Secondly, this study examines the differences in falls, disability, and mortality associated with dynapenic abdominal obesity across various measurement methods (handgrip strength, waist circumference, body mass index, and others), settings, and geographical locations, thereby offering new insights to existing research.

The findings of this study highlight the strong association between dynapenic abdominal obesity and a range of adverse health outcomes, specifically a significantly increased risk of falls, disability, and mortality among older adults. The pooled results indicate that individuals with dynapenic abdominal obesity are considerably more likely to experience falls compared to those without the condition, as demonstrated by the reported odds ratios and hazard ratios. The heightened risk of falling can be attributed to several interconnected physiological mechanisms. Dynapenia, defined as age-related loss of muscle strength independent of muscle mass, reduces lower limb function and impairs neuromuscular coordination and balance. At the same time, abdominal obesity shifts the body’s center of gravity forward, disrupts postural alignment, and compromises gait stability—factors that are particularly detrimental in older adults [22]. Furthermore, visceral fat is known to contribute to systemic inflammation and metabolic disturbances, which may exacerbate functional decline, sarcopenia, and frailty, leading to a greater risk of injury, hospitalization, and loss of independence following a fall [23]. These findings not only reinforce the clinical relevance of dynapenic abdominal obesity as a high-risk geriatric syndrome but also emphasize the need for early screening, especially in primary care and community-based settings. Incorporating routine assessments of muscle strength and waist circumference in aging populations may aid in identifying at-risk individuals. In addition, targeted interventions such as resistance training, nutritional strategies, and obesity management may help mitigate the adverse outcomes associated with dynapenic abdominal obesity and improve long-term health outcomes.

Differences in the estimation of fall risk across countries may reflect variations in assessment methods and reporting standards. In the United Kingdom, national guidelines, such as those from the National Institute for Health and Care Excellence (NICE), emphasize standardized fall history collection through structured interviews and electronic health records [24]. In Brazil, studies often rely on retrospective self-reports collected through population-based cross-sectional surveys, which may vary in consistency and be affected by recall accuracy [25]. In China, fall data are frequently derived from hospital records or caregiver-reported outcomes, which may underrepresent community-dwelling incidents [26]. In the United States, large-scale longitudinal cohort studies commonly apply validated tools, such as the Falls Efficacy Scale-International or the Morse Fall Scale, in routine follow-ups [27]. These methodological discrepancies may contribute to variations in estimates of fall prevalence and affect the comparability of studies and the pooled results of meta-analyses.

Subgroup analyses by country were conducted to explore potential geographic variations in the association between dynapenic abdominal obesity and adverse health outcomes. Our findings revealed geographic differences in fall risk, highlighting the influence of cultural, environmental, and health-care factors on fall prevalence. Tsai et al. [28] emphasized that measurement discrepancies in defining dynapenic obesity contribute to inconsistent findings in epidemiological studies, highlighting the need for standardized criteria. Moreover, Li et al. [29] indicated that regional differences in dietary patterns, physical activity levels, and health-care access substantially affect the prevalence and outcomes of obesity-related conditions, including falls. Given the limited number of studies available per country, these findings must be interpreted with caution and should primarily serve as preliminary indicators rather than definitive conclusions. Further research across diverse geographic regions is warranted to strengthen the generalizability of these findings. The association between dynapenic abdominal obesity and overall disability risk was not significant. In other words, dynapenic abdominal obesity does not increase the risk of disability. This lack of a significant association may be due to compensatory behavioral adaptations or variations in the definitions of disability across studies. Some individuals may avoid high-risk activities, reducing their exposure to scenarios that could lead to disability [14].

The current study uncovered a significant association between dynapenic abdominal obesity and increased mortality risk. The results of a subgroup analysis of measurement criteria and country of study (Italy vs. England) verified the consistency of this association across settings. This heightened mortality risk can be attributed to the compound effects of impaired muscle function and metabolic dysregulation associated with central obesity. Dynapenic abdominal obesity is linked to systemic inflammation, insulin resistance, and cardiovascular risk factors, all of which contribute to higher mortality rates [30]. Moreover, the presence of comorbidities such as diabetes and cardiovascular diseases, which are more prevalent in individuals with abdominal obesity, further exacerbates mortality risk. The meta-analysis of Smith et al. [14] highlighted the crucial role of managing abdominal obesity and maintaining muscle strength in reducing all-cause mortality among older adults. Similarly, the study of Scott et al. [31] emphasized the importance of early interventions that target muscle strength and waist circumference to mitigate long-term health risks in aging populations.

The findings of the current study indicate discrepancies in the interpretation of dynapenic abdominal obesity across studies, particularly concerning grip strength and the cutoff points for abdominal obesity. The subgroup analysis results also reveal significant differences in the risk of subsequent falls and mortality among middle-aged and older adults with dynapenic abdominal obesity assessed on the basis of different measurement methods. For example, grip strength cutoff points of <26 kg for men and <16 kg for women and abdominal obesity cutoff points of >102 cm for men and >88 cm for women [3,14] are associated with a higher risk of mortality than are other cutoff points [17,18,21]. Therefore, health-care professionals must exercise caution when selecting cutoff points for grip strength and abdominal obesity to evaluate the risk of subsequent mortality among middle-aged and older adults.

This systematic review and meta-analysis advances the current understanding of dynapenic abdominal obesity by addressing existing gaps in the literature and synthesizing its varied effects across different populations, measurement approaches, and geographic regions. We also compared our findings with those of recent studies (e.g., Tsai et al. [28]; Li et al. [8]), highlighting both consistencies and discrepancies that underscore the complexity of dynapenic abdominal obesity as a clinical entity. Clinically, these findings have important implications for healthcare professionals, particularly in supporting the early identification of dynapenic abdominal obesity and the development of targeted prevention strategies. Given its association with a range of adverse health outcomes, dynapenic abdominal obesity should be recognized as a critical geriatric syndrome that warrants integrated screening as part of routine clinical assessments. Dynapenic abdominal obesity reflects a multifactorial condition involving the interplay of muscular degeneration, metabolic dysfunction, and chronic inflammation—common processes observed in aging individuals.

These interrelated dimensions suggest that dynapenic abdominal obesity represents a distinct clinical phenotype requiring systematic evaluation in both research and clinical practice. Recent studies have strengthened the rationale for treating dynapenic abdominal obesity as an independent and clinically significant condition. For instance, Ramírez et al. [3] reported a significant association between dynapenic abdominal obesity and increased cardiovascular mortality among adults aged 50 years and older, underscoring its potential influence on long-term cardiometabolic health. Veronese et al. [15] found that dynapenic abdominal obesity was a strong predictor of multimorbidity in older adults, highlighting the importance of assessing dynapenic abdominal obesity not merely as a byproduct of aging, but as a high-risk phenotype that exacerbates multiple chronic conditions. Additionally, Oba et al. [32] revealed a significant association between dynapenic abdominal obesity and mild cognitive impairment, suggesting that its influence extends beyond physical function to neurological health. These findings emphasize the need to recognize dynapenic abdominal obesity as a clinically relevant syndrome, advocate for its inclusion in geriatric screening protocols, and prioritize further research into its multidimensional health impacts. Future studies should focus on establishing standardized diagnostic criteria and conducting longitudinal investigations to mitigate the health risks associated with dynapenic abdominal obesity.

This study has several limitations. First, the integrated analysis revealed discrepancies in the criteria used to assess dynapenic abdominal obesity, which may have introduced errors in the statistical analysis. Second, significant variation exists in the follow-up periods of the included studies, ranging from 1.5 to 14 years, potentially affecting the accuracy of risk estimates for adverse outcomes. Third, most studies adjusted for confounding variables, which may have influenced the consistency of our estimations. Fourth, given that research on the adverse effects of dynapenic abdominal obesity is relatively recent, the number of eligible high-quality studies remains modest. Fourth, variations in the selection of cutoff points for handgrip strength and abdominal obesity across studies suggest the need for uniform diagnostic criteria to assist healthcare professionals. Additionally, our ability to compare differences between countries was limited by the small number of studies from certain regions, making cross-national comparisons exploratory and inconclusive. Therefore, our conclusions should be considered preliminary, and further research with more standardized methodologies and broader geographic representation is warranted to improve the generalizability and robustness of the results.

Despite the limited number of studies included, the selected articles provided consistent and meaningful evidence regarding the adverse effects of dynapenic abdominal obesity. These findings align with those reported in recent observational studies. Importantly, all included studies were rigorously selected based on well-defined inclusion criteria and demonstrated high methodological quality, as reflected by their dynapenic abdominal obesity scores. To further address potential heterogeneity, subgroup analyses were performed, offering clearer interpretation of variations and strengthening the robustness of the results. The consistency observed across these carefully conducted studies enhances the reliability and generalizability of our conclusions and supports the overall validity of the current review. Therefore, the findings from this study are of practical relevance to healthcare professionals and may serve as a foundation for future care strategies. Clinically, these results support the need for early screening, especially in primary care settings where muscle strength and waist circumference can be routinely assessed. From a public health perspective, identifying individuals at high risk in community-dwelling populations could inform the design of fall and disability prevention programs. Furthermore, this study reinforces the importance of establishing standardized diagnostic criteria to improve comparability and generalizability in future research involving older adult populations.

## 5. Conclusions

This systematic review and meta-analysis provides strong evidence that dynapenic abdominal obesity is significantly associated with increased risks of adverse health outcomes, particularly falls and mortality, among older adults. From a clinical perspective, these findings underscore the crucial need for the early identification of dynapenic abdominal obesity and the implementation of targeted preventive strategies, particularly in primary care and community-based settings. To validate these findings, future research should focus on establishing standardized diagnostic criteria for dynapenic abdominal obesity and conducting larger prospective studies. Such efforts are essential to developing evidence-based clinical guidelines that lead to improved health outcomes for older adults.

## Figures and Tables

**Table 1:** Newcastle–Ottawa scale quality assessment of prospective cohort studies.

Category	Item	1	2	3	4	5	6	7	8	9	10	11	12
Selection	Representativeness of the exposed cohort	★	★	★	★	★	★	★	★	★	★	★	★
	Selection of the nonexposed cohort	★	★	★	★	★	★	★	★	★	★	★	★
	Ascertainment of exposure	★	★	★	★	★	★	★	★	★	★	★	★
	Outcome not present at start of study	★	★	★	★	★	★	★	★	★	★	★	★
Comparability	Comparability of cohorts on the basis of the design or analysis	★★	★	★	★★	★★	★★	★	★★	★★	★	★	★★
Outcome	Assessment of outcomes	★	★	★	★	★	★	★	★	★	★	★	★
	Was the follow-up long enough for outcomes to occur?	★	★	★	★	★	★	★	★	★	★	★	★
	Adequacy of cohort follow-up	★	★	★	★	★	★	★	★	★	★	★	★
	Total score	9	8	8	9	9	9	8	9	9	8	8	9

★ presented one score. ★★ presented two scores. Max score: 9. 1: Alexandre et al. [20]. 2: Máximo et al. [13]. 3: Dowling et al. [2]. 4: Gadelha et al. [11]. 5: Lv et al. [12]. 6: Ramírez et al. [3]. 7: Rossi et al. [17]. 8: Rossi et al. [18]. 9: Rossi et al. [21]. 10: Smith et al. [14]. 11: Veronese et al. [15]. 12: Zhang et al. [16].

**Table 2:** Included prospective cohort studies assessing dynapenic abdominal obesity and fall risk.

No.	First Author (Year)	Measurement Criteria	Population	Sample Size	Country	Sex	Age (Years)	Length of Follow-Up	OR (95% CI)	HR (95% CI)	Variables Adjusted
1	Alexandre et al. [20]	Handgrip strength <26 kg (men) <16 kg (women)	Community-dwelling	1040	Brazil	F	50	4–8 years	2.10 (0.01–0.03)	None	Socioeconomic, behavioral, and clinical characteristics and BMI
		Waist circumference >102 cm (men) >88 cm (women)									
		BMI >18.5 kg/m^2^									
2	Dowling et al. [2]	Handgrip strength <30 kg (men) <20 kg (women)	Community-dwelling	4239	England	M	60–87	2 years	2.10 (1.30–3.20)	None	Age and sex
		Waist circumference >102 cm (men) >88 cm (women)									
		BMI >18.5 kg/m^2^									
3	Gadelha et al. [11]	Handgrip strength <20.67 kg	Community-dwelling	201	Brazil	Both	60–80	1.5 years	None	3.60 (1.32–9.82)	None
		Waist circumference >88 cm (women)									
		BMI >30 kg/m^2^									
4	Lv et al. [12]	Handgrip strength <28 kg (men) <18 kg (women)	Hospital	551	China	F	65	5 years	3.39 (1.47–7.81)	None	Age, sex, marital status, education, and BMI
		Waist circumference >90 cm (men) >85 cm (women)									
		BMI ≥25 kg/m^2^									
5	Máximo de Oliveira [13]	Handgrip strength <26 kg (men) <16 kg (women)	Community-dwelling	1046	Brazil	F	60	8 years	None	2.06 (1.04–4.10)	Sex, age, floor level, polypharmacy, BMI, diabetes, joint disease, dizziness/vertigo, depressive symptoms, and functional status
		Waist circumference >102 cm (men) >88 cm (women)									
		BMI >30 kg/m^2^									
6	Smith et al. [14]	Handgrip strength <26 kg (men) <16 kg (women)	Community-dwelling	5275	Ireland	Both	63	2 years	1.80 (1.24–2.60)	None	Sex, age, education, marital status, alcohol consumption, and physical activity
		Waist circumference >88 cm (women) >102 cm (men)									
7	Veronese et al. [15]	Time >15 s in the five-time chair stand	Hospital	3844	United States	Both	40–80	9 years	1.31 (1.01–1.73)	None	None
		Waist circumference >88 cm (women) >102 cm (men)									
		BMI >18.5 kg/m^2^									
8	Zhang et al. [16]	Handgrip strength <26 kg (men) <16 kg (women)	Community-dwelling	4987	China	F	60	14 years	None	1.28 (1.02–1.60)	Age, sex, and BMI
		Waist circumference >102 cm (men) >88 cm (women)									
		BMI >30 kg/m^2^									

Note: BMI = body mass index. HR = hazard ratio. OR = odds ratio. CI = confidence interval. M = male. F = female.

**Table 3:** Included prospective cohort studies assessing dynapenic abdominal obesity and disability.

No.	First Author (Year)	Measurement Criteria	Population	Original Population	Country	Sex	Age (Years)	Length of Follow-Up	OR (95% CI)	HR (95% CI)	Variables Adjusted
1	Rossi et al. [17]	Handgrip strength <33 kg (men) <19 kg (women)	Hospital	846	Italy	Both	65–95	9 years	2.10 (1.14–3.88)	None	Age, sex, smoking habits, education, medications, and diabetes
		Waist circumference >99 cm (men) >95 cm (women)									
		BMI >30.9 kg/m^2^									
2	Rossi et al. [18]	Handgrip strength <21 kg (men) <12 kg (women)	Community-dwelling	274	Italy	Both	68–78	5.5 years	None	3.39 (1.91–6.02)	Sex and age
		Waist circumference >100 cm (men) >87 cm (women)									
		BMI >28 kg/m^2^ (men) >40 kg/m^2^ (women)									
3	Smith et al. [14]	Handgrip strength <26 kg (men) <16 kg (women)	Community-dwelling	4471	Ireland	Both	62	2 years	0.41 (0.26–0.65)	None	None
		Waist circumference >88 cm (women) >102 cm (men)									

Note: BMI = body mass index. HR = hazard ratio. OR = odds ratio. CI = confidence interval.

**Table 4:** Included prospective cohort studies assessing dynapenic abdominal and mortality.

No.	First Author (Year)	Measurement Criteria	Population	Original Population	Country	Sex	Age (Years)	Length of Follow-up	OR (95% CI)	HR (95% CI)	Variables Adjusted
1	Rossi et al. [17]	Handgrip strength <15 kg (men) <8 kg (women) Waist	Community-dwelling	262	Italy	Both	66–78	10 years	None	2.46 (1.34–4.52)	Age and sex
		circumference >100 cm (men) >87 cm (women)									
		BMI >29.3 kg/m^2^									
2	Rossi et al. [18]	Handgrip strength <33 kg (men) <19 kg (women) Waist	Hospital	846	Italy	Both	65–95	9 years	None	1.36 (0.93–1.97)	Age, sex, smoking habit, education, medications, and diabetes
		circumference >99 cm (men) >95 cm (women)									
		BMI >30.9 kg/m^2^									
3	Ramírez et al. [3]	Handgrip strength <26 kg (men) <16 kg (women)	Community-dwelling	7030	England	Both	50–77	8 years	None	1.26 (0.75–2.10)	Age, sex, schooling, total household wealth, marital status, physical activity level, smoking status, alcohol intake, diabetes, and hypertension
		Waist circumference >102 cm (men) >88 cm (women)									
		BMI ≥30 kg/m^2^									

Note: BMI = body mass index. HR = hazard ratio. OR = odds ratio. CI = confidence interval.

## Data Availability

Data are available upon request due to privacy/ethical restrictions.
